# Ablation of protein phosphatase 5 (PP5) leads to enhanced both bone and cartilage development in mice

**DOI:** 10.1038/s41419-017-0254-6

**Published:** 2018-02-12

**Authors:** Jun Wang, Yong Cao, Bin Qiu, Jianyong Du, Tingting Wang, Chao Wang, Ran Deng, Xudong Shi, Kai Gao, Zhongwen Xie, Weidong Yong

**Affiliations:** 1 State Key Laboratory of Tea Plant Biology and Utilization, Anhui Agricultural University, Hefei, Anhui China; 2 Institute of Laboratory Animal Science, Chinese Academy of Medical Sciences & Peking Union Medical College, Beijing, 100021 China; 3 Experimental Medicine Center, The First Affiliated Hospital of Sichuan Medical University, Luzhou, Sichuan, 646000 China

## Abstract

This study aimed to investigate the role of protein phosphatase 5 (PP5) on bone and cartilage development using both in vivo and in vitro approaches. Six- to 8-week- old male PP5 knockout mice (KO) and their wild-type (WT) littermate controls were randomly selected for this study, and their body weights and bone (femur) lengths were measured. Micro-computed tomography scanning (Micro-CT) was performed to determine femoral bone density and micro-architecture. Mesenchymal stem cells (MSCs) isolated from bone marrow were used to examine the effects of PP5 on osteogenesis in vitro. Whole-mount Alcian blue and Alizarin red staining were used to detect cartilage formation in newborn vertebrae, limbs, and feet. Hematoxylin and eosin (H&E) staining was performed to determine growth plate thickness. Real-time PCR analysis, western blotting, and immunohistochemistry were used to detect the expression of genes and proteins in bone marrow-derived MSCs as well as in bone and cartilage tissues. The results showed PP5 KO mice exhibited significantly reduced body weight and shorter femur length compared to WT controls. The KO mice also had significantly higher volumetric bone mineral density (BMD), trabecular bone volume, and cortical thickness in the femur. The deficiency of PP5 significantly enhanced the formation of cartilage in vertebrae, limbs, and feet. In addition, KO mice possessed a wider distal femur growth plates containing significantly more chondrocytes than WT mice. Furthermore, higher expressions of several cartilage-specific genes were observed in the articular cartilage of PP5 KO mice. Immunohistochemical labeling of growth plates demonstrated that phospho-PPARγ, Runx1, and Runx2 levels were considerably higher in the KO mice. In conclusion, PP5 is a significant negative regulator on the regulation of bone and cartilage development.

## Introduction

Mesenchymal stem cells (MSCs) are multipotent progenitors that can differentiate into a variety of cell types including fibroblasts, myoblasts, osteoblasts, chondrocytes, and adipocytes^1–3^. Differentiation and maturation of MSCs to a specific cell fate is determined by many intracellular and extracellular factors, such as secreted proteins, growth factors, hormones, genetic and epigenetic regulators, extracellular matrix molecules, and transcription factors^4–6^. These complex processes are precisely regulated by coordinated actions of multiple signaling pathways including Wnt/β-catenin, tumor growth factor-β, fibroblast growth factor, and bone morphogenetic protein pathways^7–11^. During bone and cartilage development, MSCs initially give rise to osteochondral progenitor cells, which then segregate into osteoblasts and chondrocytes in a highly controlled manner.

Previous studies have demonstrated that peroxisome proliferator-activated receptor gamma (PPARγ) has an important role in the trans-differentiation of MSCs into osteoblasts and adipoctyes^12–14^. PPARγ directly binds to the runt-related transcription factor 2 (Runx2) and inhibits its transcriptional activity and mRNA expression, resulting in the prevention of osteogenesis^15,16^. PPARγ deficiency in embryonic stem cells leads to spontaneous differentiation of MSCs into osteoblasts, but prevents their differentiation into adipocytes^17^. As MSC-derived osteochondral progenitor cells promote both osteogenesis and chondrogenesis, PPARγ might also be involved in the reciprocal regulation of chondrocyte and adipocyte development involving Runx2. Further, Runx1 is essential for chondrocyte proliferation and lineage determination^18^, suggesting that multiple Runx family proteins might be involved in the regulation of MSC fate in cartilage development. Importantly, factors that regulate PPARγ and Runx mRNA expression and protein modification might have critical roles in both bone and cartilage homeostasis.

Protein phosphatase 5 (PP5), a widely expressed serine/threonine phosphatase, has a vital role in the regulation of numerous processes including cell growth, proliferation, differentiation, migration, and survival under stress^19–21^. PP5 contains a 34-amino acid tetratricopeptide repeat motif that mediates protein–protein interaction and also serves as an auto-inhibitory domain for phosphatase activity^22,23^. Genetic studies indicated that inactivation of PP5 prevented high-fat diet feeding-induced weight gain and adipogenesis^24,25^. Several studies have demonstrated that PP5 regulates both PPARγ and Runx2 through posttranscriptional and posttranslational mechanisms^26–28^. Specifically, PP5 directly influences the phosphorylation of PPARγ (pSer-112), thereby reducing its transcriptional activity^26^. In addition, PP5 modifies the phosphorylation of Runx2 (pSer-301 and pSer-319) and influences osteoblast differentiation and activity^27^. Recently, Stechschulte et al.^29^ found that PP5 deficiency results in a significant increase in bone mass in mice, and that these mice were resistant to rosiglitazone-induced bone mass loss by regulating the phosphorylation levels of PPARγ and Runx2. These findings suggest that the activities of PPARγ and Runx2 are major factors for PP5-mediated bone development. However, it is unknown whether the PP5-mediated Runx2 and PPARγ phosphorylation similarly modulates the development of the cartilage tissue. In addition, this study is the first to investigate the role of PP5 on chondrocyte development in vivo.

In this study, we employed a genetic approach to test the effect of PP5 on bone and cartilage development in mice. We found that the formation of cartilage at different sites was enhanced, along with increases in cortical thickness and higher trabecular bone formation, in PP5 knockout (KO) mice. Further, cellular and molecular analyses revealed that PP5 deficiency resulted in higher levels of Runx1, Runx2, and phospho- PPARγ in the growth plate of articular cartilage. These increases were concomitant with the upregulation of several cartilage-specific genes in the same tissue, suggesting a common mechanism underlying PP5-mediated chondrocyte development and bone formation.

## Results

### PP5-deficient mice possess lower body weight and shorter femur length, but higher normalized femur weight

**Fig. 1:**
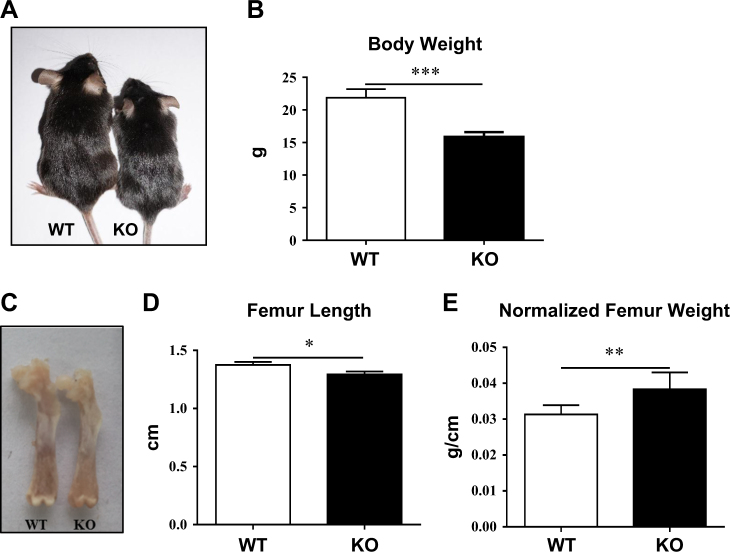
PP5-deficient mice have lower body weights and shorter femur lengths. Compared with WT mice (*n* = 15), male PP5 KO mice (*n* = 16) displayed significantly lower body weight (**a** and **b**) at 6 weeks of age. The femur length of KO mice (*n* = 6) was also significantly shorter relative to WT control mice (*n* = 6) (**c** and **d**). PP5 KO mice exhibited significantly higher femoral mass when normalized by femur length (**e**). Values are presented as mean ± SE. *P < 0.05, **P < 0.01, and ***P < 0.005 using Student’s *t-*test. KO, knockout; WT, wild-type

PP5 KO mice^30^ were previously generated using a BayGenomics promoter-trapped embryonic stem cell line XG029, which contains a 129 and C57BL/6 mixed genetic background. After more than 10 generations backcrossed with C57BL/6, PP5 KO mice were found to be significantly smaller than wild-type (WT) littermates (Fig. 1a). Compared with WT mice, male PP5 KO mice displayed 27% lower body weight (*P* < 0.01) (Fig. 1b). The femur length of KO mice was also significantly shorter (*P* < 0.05) than that of the control WT mice (Fig. 1c,d). However, when femur weight was normalized by the femur length, PP5 KO mice had significantly higher (*P* < 0.01) femoral weight compared with WT mice (Fig. 1e).

### PP5 deficiency increases trabecular bone mass and micro-architecture and improves cortical thickness

**Fig. 2:**
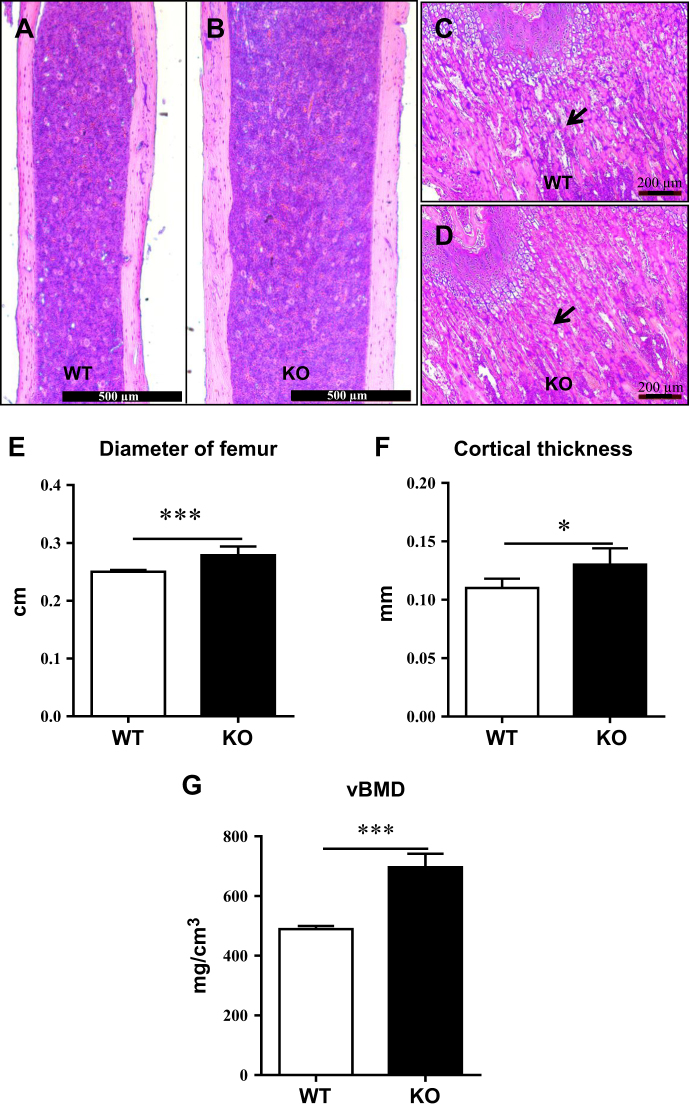
PP5 deficiency increases femur diameter, bone mass, and cortical thickness Compared with WT mice, H&E staining of femoral sections showed that PP5 KO mice had significantly thicker femurs at the diaphysis region (**a** and **b**). The amount of trabecular bone mass underneath the growth plate area of distal femur was higher in the PP5 KO mice compared to that of WT littermates (**c** and **d**). Femoral diameter and cortical thickness were significantly higher in the PP5 KO mice relative to WT (**e** and **f**). The volumetric bone mineral density (BMD) was also significantly higher in the PP5 KO mice (**g**). Values are presented as mean ± SE. *P < 0.05 and ***P < 0.005 using Student’s *t*-test. *n* = 6

**Fig. 3:**
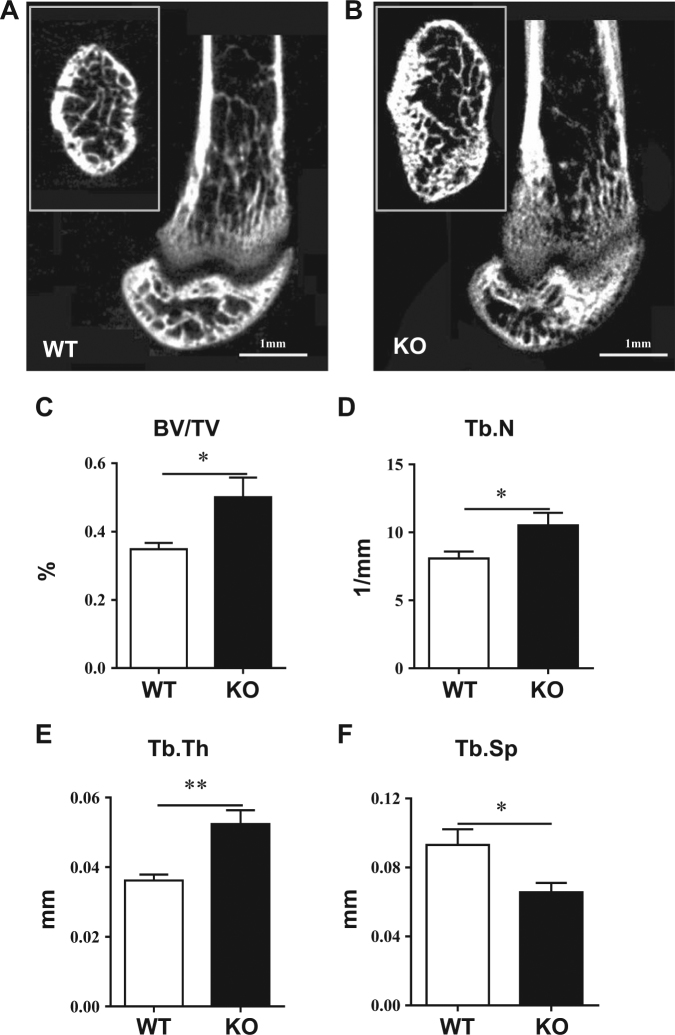
PP5 deficiency increases trabecular bone volume and micro-architecture at distal femur Micro-CT images clearly revealed improved trabecular bone volume and microarchitecture in the PP5 KO mice (**a** and **b**). Quantitative analysis demonstrated that PP5 KO mice exhibit significantly increased trabecular bone volume (BV/TV) (**c**), trabecular number (**d**), and trabecular thickness (**e**), but significantly lower trabecular separation (**f**) compared with WT control mice. Values are presented as mean ± SE. *P < 0.05 and **P < 0.01 using Student’s *t*-test. *n* = 6

To determine whether deficiency of PP5 affects bone development, we performed histological staining and micro-computed tomography (micro-CT) analysis. Hematoxylin and eosin (H&E) staining of femoral sections showed that compared to WT mice, PP5 KO mice had significantly thicker femurs at the diaphysis region (Figs. 2a,b). In addition, the amount of trabecular bone underneath the growth plate area of distal femur was higher in PP5 KO mice compared to WT littermates (Fig. 2c,d). The quantitative analysis revealed that femoral diameter and cortical thickness were significantly higher (*P* < 0.001 and *P* < 0.05, respectively) in the PP5 KO mice (Fig. 2e,f). The volumetric bone mineral density (BMD) was also significantly higher (*P* < 0.01) in the PP5 KO mice (Fig. 2g). Micro-CT images clearly revealed the trabecular bone volume differences between WT and PP5 KO mice (Fig. 3a,b). PP5 KO mice also displayed significantly increased trabecular bone volume (BV/TV) (*P* < 0.05, Fig. 3c), trabecular number (Tb.N) (*P* < 0.05, Fig. 3d), and trabecular thickness (Tb.Th) (*P* < 0.01, Fig. 3e), but significantly lower trabecular separation (Tb.Sp) (*P* < 0.05, Fig. 3f). These results indicate that deficiency of PP5 promoted bone development in KO mice.

### Ablation of PP5 enhances osteoblast differentiation and promotes osteogenesis of MSCs

**Fig. 4:**
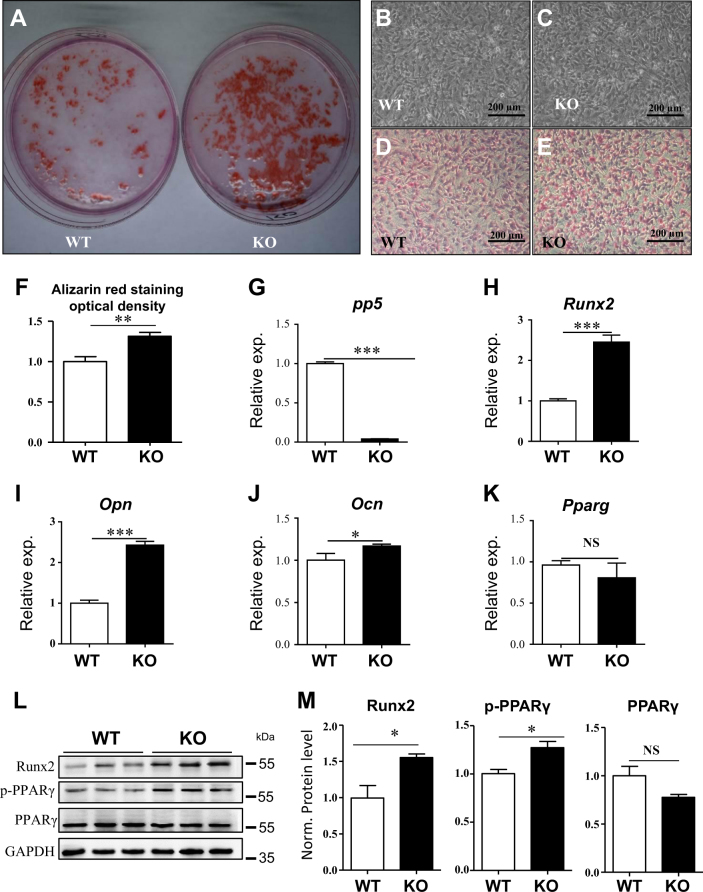
Ablation of PP5 enhances osteoblast differentiation and promotes osteogenesis of MSCs Alizarin red staining on day 14 revealed significantly higher osteoblast differentiation of MSCs in PP5 KO mice (**a**, **d**, **e**, and **f**) compared to WT mice at the same cellular density (**b** and **c**). ImageJ analysis quantified the relative Alizarin red staining optical density (**f**) based on three independent experiments. The gene expression profile of MSCs showed that in KO the mRNA expression of PP5 was significantly lower (**g**), whereas the expression of *Runx2*, *Opn*, and *Ocn* were significantly higher (**h**–**j**) relative to WT. Slightly lower expression of PPARγ was observed between in KO MSCs compared with WT cells, but failed to reach significance (**k**). Western blot analysis indicated a dramatic increase in Runx2 in PP5 KO MSCs (**l** and **m**). In addition, while total PPARγdid not differ between KO and WT cells, the expression of Phospho- PPARγ (pSER112) was higher in the KO cells (**l** and **m**). Values are presented as mean ± SE. *P < 0.05, **P < 0.01, and ***P < 0.005 using Student’s *t*-test. *n* = 3

To identify whether the ablation of PP5 could promote the osteogenesis in vitro, bone marrow MSCs were isolated from PP5 KO and WT mice. The osteoblastic differentiation of MSCs was induced in vitro and the osteoblast-specific dye alizarin red was used to stain cells on day 14 after induction. Despite possessing similar cellular densities, the osteoblastic differentiation of MSCs was higher in PP5 KO (Fig. 4a–f). These results suggested that PP5 KO MSCs had a stronger ability to differentiate into osteoblasts compared with WT MSCs. In order to investigate the effects of PP5 on bone development-related genes, we analyzed the gene expression profiles of MSCs obtained from WT and PP5 KO mice. The mRNA expression of PP5 was significantly (*P* < 0.001) decreased in KO, whereas *Runx2*, osteopontin (*Opn*), and osteocalcin (*Ocn*) were significantly increased (*P* < 0.001, *P* < 0.001, and *P* < 0.05, respectively), and no significant change in PPARγ was observed (Fig. 4g–k). Western blot (WB) analysis indicated that Runx2 is dramatically induced in PP5 KO MSCs (Fig. 4l). In addition, although the total PPARγ level did not differ between PP5 KO and WT cells, the level of Phospho-PPARγ (pSER112) was significantly higher in the KO (Fig. 4l,m).

### PP5 deficiency increases growth plate thickness and enhances cartilage development

**Fig. 5:**
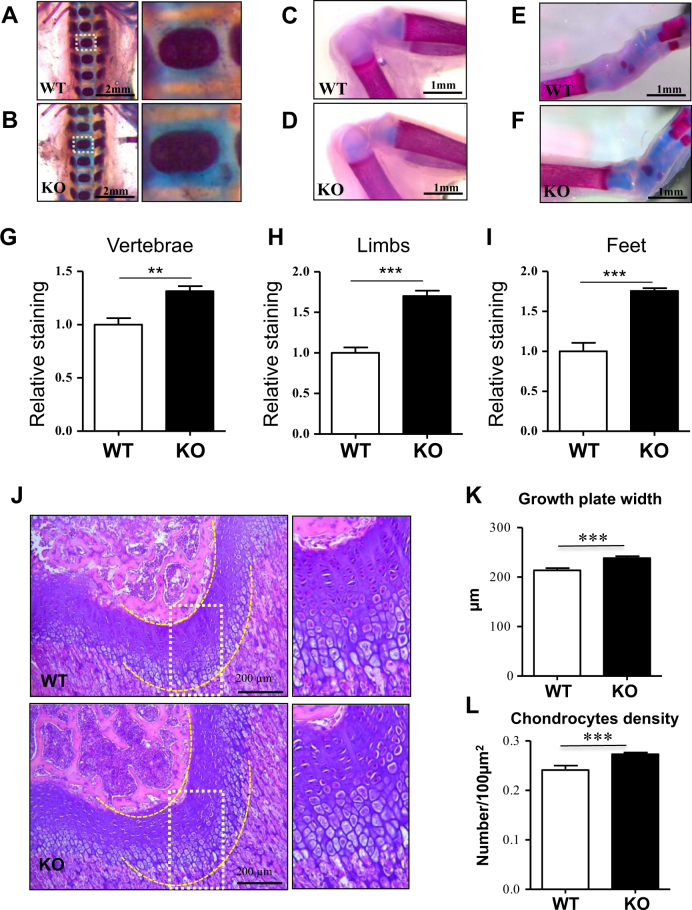
PP5 deficiency increases growth plate thickness and enhances cartilage development Whole-mount Alcian blue and Alizarin red staining of WT and PP5 KO newborn mice showed stronger blue staining in vertebrae (**a** and **b**), limbs (**c** and **d**), and feet (**e** and **f**) in the PP5 KO. Alizarin red staining was quantified by ImageJ analysis (**g**–**i**). H&E staining of femurs revealed the area of growth plate in the KO mice was wider than that of WT mice (**j**). Quantitative analyses indicated the significant increase in growth plate width (**k**) and the density of chondrocytes (cell number/100 µm^2^) within the growth plate in KO mice (**l**). Magnified areas are indicated by dashed boxes. Values are presented as mean ± SE. **P < 0.01 and ***P < 0.005 using Student’s *t*-test. Each genotype, three same positions were selected from individual H&E staining of total three animals

To determine whether PP5 influences endochondral ossification in cartilage, whole-mount Alcian blue and Alizarin red staining of newborn WT and PP5 KO mice was performed. We used newborn mice in this assay as the extent of cartilaginous tissue is greatest at this early fetal developmental stage. In vertebrae, PP5 KO mice showed stronger Alcian blue staining than WT mice (Fig. 5a,b,g), indicating that deficiency of PP5 enhanced the formation of cartilage in the spine. Similarly, PP5 KO enhanced cartilage formation in limbs (Fig. 5c,d,h) and feet (Fig. 5e,f,i) compared to WT. H&E staining of femurs further revealed a significant increase in growth plate width in KO (*P* < 0.01) (Fig. 5g,h). Quantitative analyses indicated an increase in the number of chondrocytes within the growth plate of KO as well (*P* < 0.01) (Fig. 5i). Overall, these results confirm that deficiency of PP5 affects the development of cartilage.

### KO of PP5 promotes chondrogenic proliferation and differentiation in articular cartilage

**Fig. 6:**
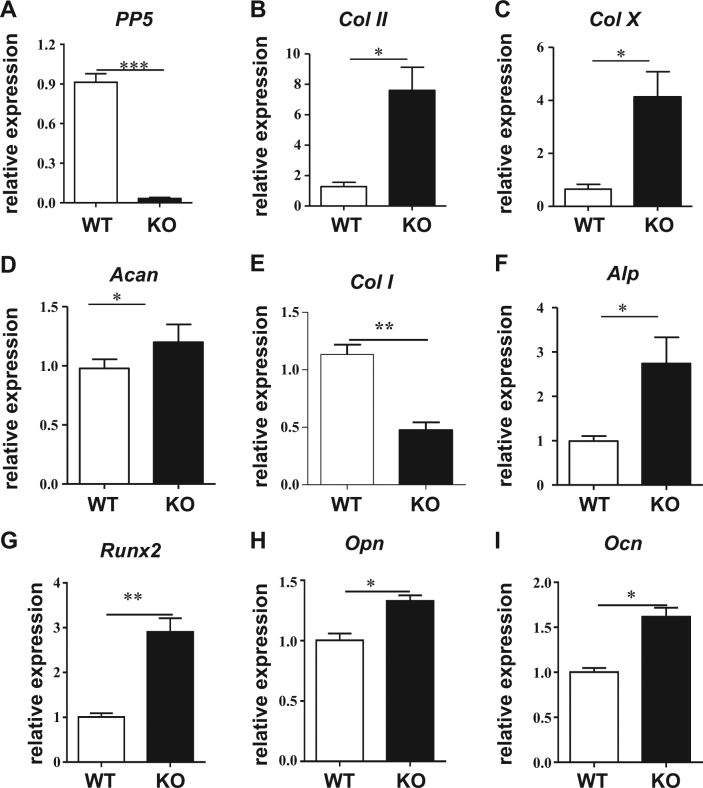
Knockout of PP5 promotes chondrogenic differentiation markers in articular cartilage. Gene expression analysis of articular cartilage from WT and PP5 KO mice indicated that the ablation of PP5 resulted in an almost 90% reduction of PP5 mRNA expression (**a**). Several chondrogenic differentiation markers such as aggrecan (*Acan*), C*ol II*, and *Col X* were significantly increased in the articular cartilage of PP5 KO mice (**b**–**d**). In addition, although mRNA expression of C*ol I* was significantly decreased (**e**), the expression of *Alp*, *Runx2, Opn*, and *Ocn* were significantly increased in the PP5 KO mice (**f**–**i**). Values are presented as mean ± SE. *P < 0.05, **P < 0.01, and ***P < 0.005 using Student’s *t-*test. *n* = 3

**Fig. 7:**
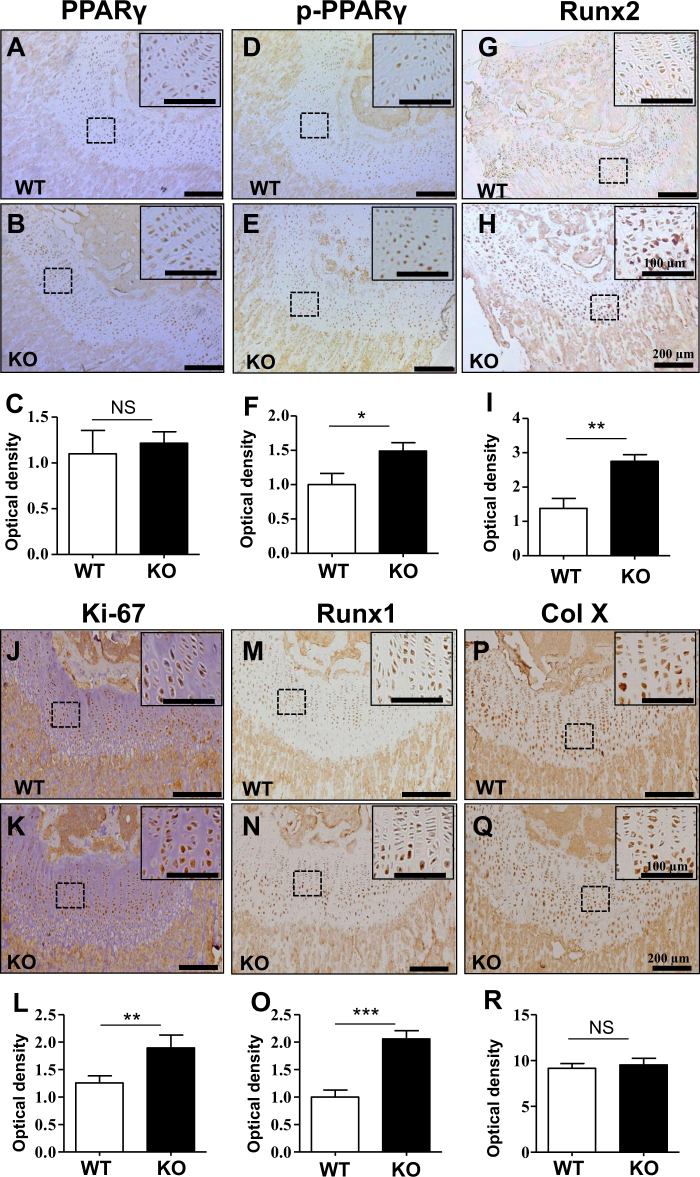
PP5 deficiency enhances chondrogenic proliferation in articular cartilage. Immunohistochemical labeling of growth plates from PP5 KO and WT mice confirmed that phospho- PPARγ (pSER112) was upregulated in KO (**d**, **e**, and **f**), whereas the level of total PPARγ remained unchanged (**a**, **b**, and **c**). In addition, Runx2 expression was markedly increased in PP5 KO (**g**, **h**, and **i**). Further, the levels of cell proliferation marker Ki-67 (**j**, **k**, and **l**) and chondrocyte proliferation-related protein Runx1 (**m**, **n**, and **o**) was increased in the growth plates of PP5 KO mice relative to that of WT (bar = 200 µm). However, Col X (**p**, **q**, and **r**) showed no significant change. Magnified areas are indicated by dashed boxes (bar = 100 µm). Quantitative analyses (**c**, **f**, **i**, **l**, **o**, and **r**) were based on optical density of immunohistochemical staining using NIH ImageJ software. Values are presented as mean ± SE. *P < 0.05, **P < 0.01, and ***P < 0.005 using Student’s *t-*test. Each genotype, at least five areas of same samples were selected from individual staining and analyses

To investigate the effects of PP5 on cartilage development-related genes, we analyzed the gene expression profiles of articular cartilage from WT and PP5 KO mice. The ablation of PP5 caused an almost 90% reduction (*P* < 0.005) of PP5 mRNA expression in the articular cartilage (Fig. 6a). Analyses of several chondrogenic differentiation markers revealed that aggrecan (*Acan*), *Col II*, and *Col X* were significantly increased (*P* < 0.05) in the articular cartilage of PP5 KO mice (Fig. 6b–d). In addition, mRNA expression of *Alp* (*P* < 0.05), *Runx2* (*P* < 0.01), Opn (*P* < 0.05), and *Ocn* (*P* < 0.05) were increased in the PP5 KO mice compared with WT controls (Fig. 6f–i). These results indicate that the absence of PP5 not only promotes osteogenesis, but also enhances cartilage development in mice. Further, immunohistochemical labeling of growth plates demonstrated that Runx2 and Runx1 expressions are markedly increased in KO (Fig. 7h,I,m,n,o). In agreement with the WB results, no difference in total PPARγ expression was observed (Fig. 7a,b,c), but phospho- PPARγ (pSER112) labeling was upregulated in KO mice (Fig. 7e,f). In addition, the levels of the cell proliferation marker Ki-67 (Fig. 7j–l) and the chondrocyte proliferation-related protein Runx1 (Fig. 7p–r) were increased in the PP5 KO, indicating that the absence of PP5 resulted in greater chondrocyte proliferation and differentiation in the growth plate.

## Discussion

The present study demonstrated that PP5 KO mice have significantly reduced body weight and shorter femur length compared with WT controls. Micro-CT measurements revealed significantly higher volumetric BMD, trabecular bone volume, and cortical thickness in the KO mice, indicating that PP5 negatively influences the bone growth and development in mice. The whole-mount Alcian blue and Alizarin red staining of newborn mice showed that deficiency of PP5 significantly enhances the formation of cartilage invertebrae, limbs, and feet. In addition, the area of the growth plate at the distal femur in KO mice is substantially wider than WT controls and the number of chondrocytes in the growth plate of KO mice is markedly higher than in WT mice. Further, higher expressions of several cartilage-specific genes were observed in the articular cartilage of PP5 KO mice. Together, these results indicate for the first time that PP5 might have a critical role in cartilage development and further supports its role in the regulation of bone homeostasis.

We previously demonstrated that the level of PPARγ phosphorylation (Ser-112) is elevated in the embryonic fibroblasts from PP5 KO mice^26^. In this study, we observed that the level of p-PPARγ (Ser-112) was increased along with unchanged PPARγ mRNA expression in the femurs of PP5 KO mice. It was previously shown that PP5 shifts cell lineage translocation towards adipogenesis directly by the modification of PPARγ (Ser-112) phosphorylation and through regulation of the transcriptional activity of PPARγ^26^. Recently, Stechschulte et al.^29^ found that PP5 deficiency leads to a significant increase in bone mass in mice and these mice were resistant to loss of bone mass induced by rosiglitazone through regulating the phosphorylation levels of PPARγ and Runx2. In the current study, we found that the expression of *Runx2* was significantly increased in the bone tissues of PP5 KO mice and although total PPARγ did not show significant changes, the expression of S112 of PPARγ is increased in this genotype. Further, expression of target genes downstream of Runx2, such as *Opn* and *Ocn*, were significantly increased in the same tissues in KO mice. WB analysis also revealed an increased abundance of Runx2 protein in the KO femur. Using in-vitro studies, Stechschulte et al. demonstrated that shRNA inhibition of PP5 expression in U-33/γ2 cells isolated from bone marrow resulted in higher levels of PPARγ Ser-112 and Runx2 Ser-319 phosphorylation compared with control cells^29^. Overexpression of PP5 in these cells also led to lower transcriptional activity of Runx2 and higher transcriptional activity of PPARγ compared with the control cells^29^. Our in-vitro data in the current study using MSCs derived from bone marrow of littermate WT and KO also show that osteoblast differentiation is significantly increased in cells from PP5 KO compared with WT. Together, these results provide further verification that PP5 has a significant effect on bone tissue development mediated through PPARγ and Runx2 phosphorylation.

The whole-mount Alcian blue and Alizarin red staining of newborn PP5 KO mice revealed stronger Alcian blue staining in vertebrae, limbs, and feet compared with WT mice (Fig. 5a–f), suggesting that deficiency of PP5 enhances the formation of cartilage at these locations. H&E staining of femurs further revealed that the area of the growth plate at the distal femur in KO mice (Fig. 5g,h) is substantially wider compared with the WT mice and that the number of chondrocytes within the growth plate of KO mice is higher than in WT controls (Fig. 5i), indicating that deficiency of PP5 positively influences the development of cartilage. Moreover, when gene expression profiles were analyzed using articular cartilage from PP5 KO and WT mice, significantly higher levels of expression of several cartilage-specific genes (*Col II*, *Col X*, and *Acan*) were observed in the KO mice (Fig. 6b–d). *Runx2* mRNA expression was significantly increased (Fig. 6g), whereas *Col I* was significantly decreased (Fig. 6e) in the cartilage of PP5 KO mice. In addition, Runx2 downstream target genes (*Alp*, *Opn*, and *Ocn*) were significantly elevated in the cartilage of KO mice (Fig. 6f,h,i). These results suggest that gene expression plays a significant role in PP5-mediated cartilage development in mice. Moreover, immunohistochemical labeling revealed a higher expression of PPARγ Ser-112 and Runx2 proteins in the growth plate of PP5 KO mice (Fig. 7g–i). Runx1, a chondrocyte proliferation and lineage determinant factor, and Ki-67, a cell proliferation marker, were increased in the KO growth plate (Figs. 7m–o). Collectively, these results suggest that PP5 deficiency has significant positive effects on chondrocyte development in mice.

The Runx family of transcription factors have been shown to play a crucial role in chondrocyte differentiation^18,31^. Runx1 is present during mesenchymal condensations, in resting and proliferating zones of chondrocytes, and also in mature chondrocytes^32^. In mesenchymal-specific Runx1 KO mice, mesenchymal cells condense normally but have delayed commitment to the chondrocyte lineage^18^. In addition, mice deficient for Runx1 in non-hematopoietic lineages develop normal skeletons but present with delayed endochondral development of sternal vertebrae, non-fusion of the supraoccipital bone, and failure of sternal mineralization^31^. A recent article demonstrated that Runx1 promotes cell proliferation in the superficial zone of chondrocytes^32^. Runx2 is an indispensable key transcription factor in the process of osteogenesis^33^. Runx2 also directly induces non-hypertrophic differentiation of chondrocytes^34^. The KO of *Runx2* gene in mice results in a reduction in the number of mature chondrocytes and osteoblasts, leading to failure of bone formation and death of mice shortly after birth^35^. In contrast, overexpression of *Runx2* in chondrocytes induces ectopic chondrocyte hypertrophy in mice^34^. In addition, Runx2 activates the expression of important chondrogenic and osteogenic marker genes including *Alp*, *Col I, Bsp*, *Opn*, and *Ocn* by binding to osteoblast-specific element 2 on the promoter of these genes^33,36,37^. Importantly, the current study shows that PP5 significantly influences the upregulation of Runx1 and Runx2 by increasing the phosphorylation of PPARγ Ser-112 and reducing the transcriptional activity of PPARγ, suggesting that both members of the Runx family of transcription factors play crucial roles during bone and cartilage development.

In this study, we observed higher mRNA expression of Aggrecan (*Acan*) in the articular cartilage in PP5 KO mice. Aggrecan is a critical proteoglycan component of the extracellular matrix of growth plates and articular cartilage^38^. Heterozygous mutations in the *Acan* gene cause autosomal dominant short stature with articular cartilage dysfunction in humans^39^. Aggrecan is required for growth plate cytoartchitecture and differentiation and it has been suggested that this molecule might have a major role in regulating the expression of key growth factors and signaling molecules during cartilage development^40^. In addition, cartilage-specific PPARγ KO mice exhibit higher expression of *Acan*, suggesting PPARγ might be a regulator of cartilage formation through the modulation of aggrecan^41^. Wigner et al.^42^ reported that Runx factors may combine with promoter of *Acan* to regulate its transcription, suggesting that Runx1 might influence chondrocyte proliferation by interacting with the promoter of *Acan*. The current study also indicated that the expression levels of *Acan* and *Runx1* were significantly increased in the articular cartilage in PP5 KO mice. Together, these results suggest that through the regulation of Aggrecan, PPARγ and Runx1/2, PP5 might act as a significant contributor for the development and growth of cartilage tissue.

This study has several limitations. We investigated bone phenotypes in 6–8-week-old mice and cartilage phenotypes in mice at newborn and 6–8 weeks of age. In addition, we used only male mice in this study. Further, we did not examine the influence of PP5 deficiency on other systems of the body such as muscle and fat tissues in this study. Thus, the influence of PP5 on bone and cartilage development in mice as they age needs to be further explored. Moreover, whether and how sex hormones contribute to the bone and cartilage homeostasis in the presence of PP5 deficiency need further investigation.

In conclusion, this study demonstrated that deficiency of PP5 enhances the formation of cartilage at multiple sites including vertebrae, limbs, and feet. In addition, the absence of PP5 led to wider growth plates, higher numbers of chondrocytes, and higher expressions of several cartilage-specific genes, suggesting that PP5 has significant effects on cartilage development in addition to its known role in bone formation.

## Materials and Methods

### Experimental animals

PP5 KO mice^30^ and littermates were bred on a C57BL/6 genetic background. All animal experiments were conducted in accordance with “Guide for the Care and Use of Laboratory Animals” and were approved by Animal Care and Research Advisory Committee in the Institute of Laboratory Animal Sciences, Chinese Academy of Medical Sciences.

### BMD and micro-architecture measurements

Male WT and PP5 KO mice (*n* = 6 each genotype) at 6–8 weeks of age were killed with carbon dioxide and the lower limbs (femur and tibia) were dissected from these animals. The femurs were scanned using PIXImus CT (Siemens, Germany) and measured for volumetric BMD (mg/cm^3^), BV/TV, and micro-architectural parameters (Tb.N, Tb.Th, Tb.Sp, and cortical bone thickness (Ct. Th)) using Inveon Research Workplace II software (Germany). In addition, Ct. Th and femur diameter were determined at the femoral midshaft.

### Alican blue and Alizarin red staining

Newborn WT and PP5 KO mice (*n* = 3 each genotype) were killed and skins were removed from these animals. After fixation in 95% ethanol overnight, whole-mount Alcian blue and Alizarin red staining was performed as described previously^11^. Images of the lower limbs, feet, and vertebrae were obtained using an optical microscope. Optical density measurements have been performed using NIH ImageJ software.

### Cell culture and osteogenic differentiation in vitro

MSCs were isolated from PP5 KO and WT mice following the protocol described in ref. ^25^ with a slight modification. MSCs were plated in a 60 mm cell culture dish at a density of 3 × 10^5^ cells/ml. When the cells reached a confluence of 90%, the medium was replaced with osteogenic medium (α-minimum essential medium containing 10% fetal bovine serum, 5 µg/ml ascorbic acid, and 10 mM β-glycerophosphate). The medium was replaced every 2 days until day 14, when differentiated MSCs were stained using Alizarin Red S (Sigma-Aldrich, St. Louis, MO, USA). Optical density measurements have been performed using NIH ImageJ software.

### RNA isolation and quantitative real-time PCR analysis

Total mRNA was isolated from bone and cartilage tissues (from three male WT and three male PP5 KO mice at 6 weeks of age) and MSCs cells using Trizol reagent (Life Technologies, Gaithersburg, MD, USA). Subsequently, purification of total mRNA was performed using an mRNA purification kit (TransGen Biotech, Beijing, China). One microgram of purified total mRNA was used to synthesize cDNA using PrimeScript RT Reagent Kit (TaKaRa Biotechnology Co. Ltd, Dalian, China). Real-time qPCR was performed using SYBR® Premix Ex Taq II (TaKaRa Biotechnology Co, Ltd) and Applied Biosystems 7500 Real-Time PCR System (ABI, Waltham, MA, USA). Glyceraldehyde 3-phosphate dehydrogenase (*Gapdh)* was used as a housekeeping gene and expression level was presented as fold change relative to the control. All primers were synthesized by Invitrogen (Beijing, China) and are listed in Table 1.

**Table 1:** Primer sequence information used for this study

Primers name	Primer sequence
*Ocn*	F:5′−GACCTCACAGATGCCAAGCCC−3′
	R:5′−ATAGATGCGTTTGTAGGCGGTC−3′
*Opn*	F:5′−AACCAGCCAAGGACTAACTACG−3′
	R:5′−AAGCTTCTTCTCCTCTGAGCTG−3′
*Runx2*	F:5′−CATTTGCACTGGGTCACACGTA−3′
	R:5′−GAATCTGGCCATGTTTGTGCTC−3′
*Colla1*	F:5′−CAACCTCAAGAAGTCCCTGC−3′
	F:5′−AGGTGAATCGACTGTTGCCT−3′
*PPAR*γ	F:5′−GGGTGAAACTCTGGGAGATT−3′
	R:5′−ATGCTTTATCCCCACAGAC−3′
*PP5*	F:5′−AACAAGATCGTGAAGCAGAAGGCC−3′
	R:5′−TTCGTGGCTGCGGATGATATAGTC−3′
*GAPDH*	F:5′−ATCAACGGGAAGCCCATCAC−3′
	R:5′−TTGGCTCCACCCTTCAAGTG−3′

### Histology

Femurs of PP5 KO and WT mice (*n* = 3 each genotype) at 6–8 weeks of age were fixed in 10% neutral buffered formalin solution for 48 h, then decalcified in 0.3 M EDTA until the femurs could be penetrated by a needle. Subsequently, femurs were dehydrated with gradually increasing concentrations of ethanol, embedded in paraffin, and sectioned at 5 μm thickness. After paraffin was cleared by 100% xylene, the femoral slices were stained with H&E.

### Immunohistochemistry and WB analysis

Immunohistochemical labeling of femur slices was performed as previously described^43^ using the following antibodies: PP5 (SC-67039, Santa Cruz, USA), Runx1 (ab23980, Abcam, England), Runx2 (ab192256, Abcam), PPARγ (ab45036, Abcam), Phospho- PPARγ (pSER112) (SAB4503977, Sigma, USA), Ki67 (ab15580, Abcam), and ColX (ab58632, Abcam). Images were obtained under an optical microscope (DM600B, Leica, Germany). WB analysis was performed with standard procedures using the same antibodies used for immunohistochemistry. WB signals were captured by Tanon 5500 Chemiluminescent Imaging System (Tanon, Shanghai, China). Optical density was quantified using NIH ImageJ software.

### Statistical analysis

Quantitative data were represented as mean ± SE. Statistical differences between WT and PP5 KO mice were compared by independent Student’s *t*-test using GraphPad Prism. *P* ≤ 0.05 indicated a statistically significant difference.
